# Assessing the Significance of Hyperthermia in Newborns Undergoing Phototherapy for Hyperbilirubinemia

**DOI:** 10.1055/a-2642-7488

**Published:** 2025-07-11

**Authors:** Krishna Trivedi, Janet D.-Williams, Rohan Rao, Allison Rometo, Benjamin Miller

**Affiliations:** 1 Department of Pediatrics Northwestern University Feinberg School of Medicine, Chicago, Illinois; 2 Department of Pediatrics, University of Pittsburgh School of Medicine, Pittsburgh, Pennsylvania

**Keywords:** neonate, fever, phototherapy, hyperbilirubinemia, serious bacterial infection, hyperthermia evaluation

## Abstract

**Objective:**

About 2% of full-term neonates are evaluated for fever, with serious bacterial infections (SBIs) identified in roughly 10% of cases. The 2021 American Academy of Pediatrics guideline standardizes febrile neonate evaluation, but factors like phototherapy for hyperbilirubinemia can complicate decisions. Phototherapy-associated hyperthermia raises concern about distinguishing environmental causes from true infection. This study assessed the prevalence of hyperthermia in neonates receiving phototherapy and its association with SBI.

**Study Design:**

We performed a retrospective chart review of neonates admitted for phototherapy at a quaternary pediatric hospital (2019–2022). Using International Classification of Diseases codes, we identified patients with hyperthermia (≥38°C) and reviewed whether they underwent SBI evaluation and follow-up within 2 weeks.

**Results:**

Among 639 neonates, 9 (1.4%) developed hyperthermia. Two (22%) were diagnosed with an SBI; one had a negative SBI workup, and six were not further evaluated. None of the seven without SBI returned for care. The 1.4% hyperthermia rate is not higher than the general neonatal fever prevalence (2%).

**Conclusion:**

Hyperthermia during phototherapy is uncommon, but the 22% SBI rate in febrile neonates is noteworthy. Elevated temperatures in this context should not be presumed to be environmental. Clinicians should maintain vigilance and consider full SBI evaluations.

**Key Points:**

- Consider SBIs in febrile neonates on phototherapy; do not attribute fever to environmental factors.
- Phototherapy rarely causes fever; while it warms infants, it does not raise fever risk.
- Standardized approaches reduce variability in fever care.

Approximately 2% of full-term neonates are evaluated for fever in the neonatal period, with variable approaches in the evaluation and care of these patients. 1 2 Of these patients, the prevalence of life-threatening serious bacterial infections (SBIs) has remained approximately 10% for more than 30 years. 1 Though the 2021 American Academy of Pediatrics Clinical Practice Guideline for the evaluation and treatment of well-appearing febrile neonates provides a means of decreasing practice variability, confounding factors that cause hyperthermia can lead to inconsistencies in management.

One potential confounding factor is phototherapy for neonatal hyperbilirubinemia. Given the nuances of isolette temperature regulation and perception of the phototherapy/isolette environment leading to higher temperatures, some providers question whether hyperthermia during phototherapy is due to environmental factors or underlying infection. This adds variability in the evaluation and management of patients who have a fever while undergoing phototherapy. Our study aimed to assess the prevalence of hyperthermia in neonates undergoing phototherapy for hyperbilirubinemia and the significance of hyperthermia as it relates to SBI in this population.

## Methods

Through retrospective chart review at a single, quaternary care pediatric institution, we extracted temperature measurements from the electronic medical records (EMRs) of neonates admitted to acute care units for hyperbilirubinemia requiring phototherapy over a 4-year period from 2019 to 2022. Detailed chart review of neonates with temperature ≥38°C revealed when further evaluation was conducted and the outcomes of any such evaluation. From these data, we calculated the prevalence of hyperthermia in neonates who were undergoing phototherapy and the prevalence of SBI in this population. Patients of interest for the study were identified using relevant International Classification of Diseases (ICD) codes for hyperbilirubinemia requiring phototherapy. Chart review included data gathered for that admission as well as a review for possible readmissions over the subsequent 2 weeks to capture any patients that may have had SBI during this time. The study was approved by our Institutional Review Board.

## Results

**Fig. 1:**
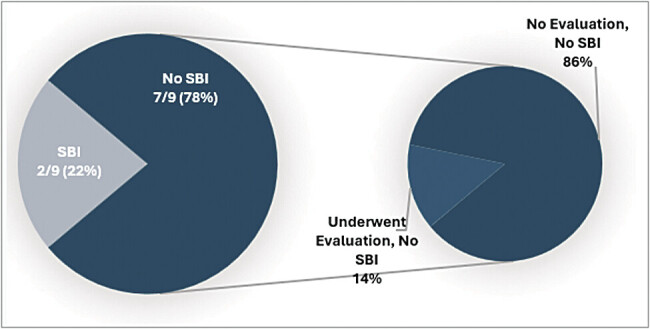
Evaluation of patients with hyperthermia during admission for phototherapy.

During the study period, 639 infants were admitted to an acute care bed with the diagnosis of hyperbilirubinemia requiring phototherapy. Nine (1.4%) of these infants had recorded temperatures ≥38°C. Of these, two (22%) had an SBI. For the other seven febrile patients, one underwent evaluation for a potential SBI, which was negative. None returned for care within 2 weeks following discharge. Details of these patients are seen in Table 1 and Fig. 1 .

**Table 1:** Patients noted to have temperature ≥38°C while admitted for phototherapy for hyperbilirubinemia, associated documentation, evaluation course, and clinical outcome

Patient	Age at presentation (d)	Maximum temperature reading	Hyperthermia noted in provider documentation?	SBI evaluation performed?	Patient outcome	Provider rationale in documentation (when hyperthermia is noted)
A	4	38.0	Yes	No	Returned to care 2 d after index discharge with E. coli sepsis/meningitis	She had an elevated temperature while admitted under phototherapy, tmax of 100.4, which resolved after bili lights were discontinued. Subsequent temps were all within normal limits. No sepsis workup was initiated at this time, as this was thought to be environmental, and no antibiotics were given. ANC on admission was <4,000 with no notable WBC elevation
B	5	38.0	Yes	Yes	E. coli bacteremia, treated effectively without adverse outcome	
C	3	38.2	No	No	No further fever and no return for care after discharge	
D	3	38.0	Yes	No	No further fever and no return for care after discharge	One temperature up to 38.0, but occurred when in an isolette, under phototherapy. Upon repeat, her temperature decreased to 37.3 within 30 min without intervention, so a rule-out sepsis evaluation was not performed
E	3	38.0	No	No	No further fever and no return for care after discharge	
F	4	38.0	Yes	Yes	Blood, urine, and CSF cultures were all negative. No return for care after discharge	
G	5	38.1	No	No	No further fever and no return for care after discharge	
H	5	38.3	No	No	No further fever and no return for care after discharge	
I	7	38.1	No	No	No further fever and no return for care after discharge	

## Discussion

In our cohort, the prevalence of hyperthermia in patients undergoing phototherapy was 1.4% and relatively low. Compared with the previously reported 2% suggesting no increased risk for hyperthermia when undergoing phototherapy. Most noteworthy, in our study, the prevalence of SBI in febrile neonates (22%) receiving phototherapy exceeded that of the general febrile neonate population published previously. 1

Given similar or even lower rates of fever in neonates undergoing phototherapy, and higher prevalence of SBI we observed in our data, we believe that the environmental impacts of phototherapy should not be used as an explanation for hyperthermia in this patient population and that these patients should undergo further SBI evaluation.

Limitations of our study include only a single center and a relatively small sample size. Our patient data was pulled using ICD codes for hyperbilirubinemia requiring phototherapy, but may be impacted by variabilities in primary diagnosis codes, possibly leading to patients not being included in our data. Additionally, it is possible that patients treated within our system for hyperbilirubinemia may have been admitted with an SBI to another facility and, therefore, not accessible in our EMR.

Many guidelines for the evaluation and management of hyperthermia in neonates include hyperbilirubinemia as a risk factor for underlying sepsis. Therefore, the presence of hyperthermia and hyperbilirubinemia deserves further evaluation for an underlying infectious etiology.
